# Fatal Adverse Events Associated With Programmed Cell Death Ligand 1 Inhibitors: A Systematic Review and Meta-Analysis

**DOI:** 10.3389/fphar.2020.00005

**Published:** 2020-01-31

**Authors:** Xuewen Wang, Shijie Wu, Yaying Chen, Erqian Shao, Tingting Zhuang, Linbin Lu, Xiong Chen

**Affiliations:** ^1^ Department of Oncology, The 900^th^ Hospital of the People’s Liberation Army Joint Service Support Force, Fuzong Clinical Medical College of Fujian Medical University, Fuzhou, China; ^2^ Department of Clinical Medicine, Fujian Medical University, Fuzhou, China

**Keywords:** PD-L1 inhibitors, fatal adverse events, incidence, meta-analysis, interstitial lung disease

## Abstract

**Purpose:**

We performed this systematic review and meta-analysis to assess the incidence of fatal adverse events that were associated with the use of programmed cell death ligand 1 (PD-L1) inhibitors, to describe them and to statistically depict factors that were associated with these events.

**Method:**

PubMed, Embase, and Cochrane Library were completely searched based on the following terms or relevant Medical Subject Heading ones: “atezolizumab”, “durvalumab”, “avelumab”, and “cemiplimab”.

**Results:**

A total of 26 eligible studies were identified, incorporating 6,896 unique participants. The overall incidence was 1.24% (95% CI: 0.93–1.65%). The incidence and odds were higher in patients with non-squamous non-small cell lung cancer (NSCLC) than those with urothelial carcinoma [(2.25 vs. 0.85, *p =* 0.04), (odds ratio [OR]: 2.69; 95% CI: 1.04–6.97, *p =* 0.04)], higher in the middle-aged group than the young group [(1.74 vs. 0.89, *p =* 0.01), (OR: 2.13; 95% CI: 1.26–3.61, *p =* 0.01)], and higher in the trial phase I than the trial phase II [(1.76 vs. 0.60, *p =* 0.01), (OR: 0.31; 95% CI: 0.13–0.75, *p =* 0.01)]. Notably, the trial phase I had a higher incidence than trial phase II or III following regulating for cancer types and average age (OR: 0.28; 95% CI: 0.11–0.71, *p =* 0.01, OR: 0.48; 95% CI: 0.24–0.95, *p =* 0.04, respectively). In terms of organ-specific fatal adverse events, interstitial lung disease (ILD) was frequently documented. A variety of respiratory system-related fatal adverse events were recorded, including but not limited to pneumonia and respiratory failure. As for organ-unspecific fatal adverse events, substantial cases of sepsis and neutropenia were recorded.

**Conclusion:**

This study firstly provided a comprehensive incidence and the spectrum of fatal adverse events associated with PD-L1 inhibitors, and identified three potential susceptible factors of that, yielding a capability for clinicians to distinguish high-risk populations from relatively low-risk ones, and facilitating to improve the safety of PD-L1 inhibitors broadly used in the clinical setting.

## Introduction

Numerous deaths are triggered by malignant carcinoma annually, which was accounted for 22% of all deaths in 2016 in the United States (Siegel et al., 2019). Immunotherapy, enhancing antitumor immune responses, has provided a new weapon for treating cancer in recent years. Immune checkpoint inhibitors (ICIs), including cytotoxic-T-lymphocyte-associated antigen 4 (CTLA-4), programmed cell death 1 (PD-1) and whose ligand termed programmed cell death ligand 1 (PD-L1), have been approved as therapeutic regimens for various cancers. Of that PD-L1 inhibitors have been confirmed that it could significantly improve the overall survival (OS) and progression-free survival (PFS) of patients with a range of cancers (Brahmer et al., 2012; Rittmeyer et al., 2017). The notable effect of PD-L1 inhibitors indicated it might become the essential component of ICI combination regimens in the future (Kyi and Postow, 2016). Regardless of these benefits, several studies revealed that fatal adverse events associated with PD-L1 inhibitors occasionally emerged in some patients (Barlesi et al., 2018; Choueiri et al., 2018).

The prior studies demonstrated that the common PD-L1 inhibitors-related adverse events were fatigue and diarrhea. The incidence of grade 3 or 4 toxic effects that regarded as related to PD-L1 inhibitors was 9% (Brahmer et al., 2012). Additionally, although rare, previous studies suggested that cardiac toxicity related to PD-L1 inhibitors also occurred in several patients (Varricchi et al., 2018). There were several articles regarding PD-L1 inhibitors reported immune-related adverse events (irAEs), whereas a majority of studies focused on general toxicity instead of lethal toxicity. Regrettably, we have limitedly known about the incidence, the spectrum and susceptible factors of PD-L1 inhibitors-related fatal adverse events until now. Given the increased employment of PD-L1 inhibitors in the clinical setting, the fatal adverse events associated with PD-L1 inhibitors were regarded as a crucial field to be profoundly considered.

Thus, we performed this systematic review and meta-analysis to assess the incidence of fatal adverse events that were associated with the use of PD-L1 inhibitors, to describe them and to statistically depict factors that were associated with these events.

## Methods

### Data Source

PubMed, Embase, and Cochrane Library were completely reviewed according to the following terms and relevant Medical Subject Heading ones: “atezolizumab” or “durvalumab” or “avelumab” or “cemiplimab” before July 15, 2019.

### Selection Criteria

The inclusion criteria were listed as follows: (1) the study regarding PD-L1 inhibitors therapy, including atezolizumab, durvalumab, avelumab, and cemiplimab; (2) the availability of fatal adverse events; (3) researches were published in English. In addition, the exclusion criteria were as below: (1) the unavailability of fatal adverse events associated with PD-L1 inhibitors; (2) subgroup analyses from other studies; (3) researches were not published in English.

### Data Extraction

The following data were extracted from each eligible study: the kind of PD-L1 inhibitors, cancer types, the trial phase of studies, the PD-L1 status, the race, the gender, the treatment line, the average age, and the sum of patients as well as the amount and type of fatal causes. Lung cancer was separated as the following three groups: (1) non-small cell lung cancer (NSCLC) group; (2) non-squamous NSCLC group; (3) small cell lung cancer (SCLC) group. Besides, the treatment line was divided into two components: (1) first-line treatment group, representing patients never receiving any treatment; (2) prior treatment group, representing patients have been undergone systemic treatment. To ensure the number of studies in different age groups sufficient for subgroup analysis, which could improve the reliability of statistical results, the average age of patients was divided into three categories as follows: (1) young group, including the patients with age between 55 and 62; (2) middle-aged group, comprising the patients with age between 63 and 67; (3) old group, incorporating the patients with age≥67. Additionally, the death of patients was merely attributed to PD-L1 inhibitors rather than disease progression or other drugs, which was termed as fatal adverse events associated with PD-L1 inhibitors. To standardize the terminology applied in various articles, the same type of disease would be described in uniform terms. Pneumonitis was classified as interstitial lung disease (ILD). Acute hypoxic respiratory failure and acute respiratory failure were described as respiratory failure. Also, ileus was categorized as intestinal obstruction.

Evaluation of eligible studies and extraction of data were accomplished individually by two reviewers following the Preferred Reporting Items for Systematic Reviews and Meta-Analyses (PRISMA) guidelines (Liberati et al., 2009). The discrepancy was addressed by other researchers.

### Statistical Methods

The study was aimed to analyze the incidence of the rare event. Consequently, to improve the credibility of the results, the raw data was conformed to a normal distribution by logit transformation (Barendregt et al., 2013). Mixed-effects logistic regression was performed to determine the overall incidence and corresponding 95% confidence intervals (CI) of fatal adverse events associated with PD-L1 inhibitors. The subgroup analyses were conducted according to the kind of PD-L1 inhibitors, the type of cancer, the trial phase of studies, the PD-L1 status, the race, the gender, the treatment line, and the average age of patients (*P* < 0.05 suggested statistical significance) to estimate the incidence of different classifications. The heterogeneity was judged by Higgins inconsistency index (*I* ^2^) test whose value higher than 50% implied substantial heterogeneity (Higgins and Thompson, 2002). The univariate meta-regression analyses were conducted to estimate the relationship between different covariates and the incidence of fatal adverse events associated with PD-L1 inhibitors and provide potential predictors. The multivariate meta-regression analyses were conducted to identify key predictors, including variables that displayed in univariate meta-regression analyses with a value of *p* < 0.05 and that were regarded as clinically relevant. Given the number of samples, we would carefully select eligible variables to enroll in the multivariate meta-regression analyses. The funnel plot and Egger tests were used to estimate the publication bias of this study (Egger et al., 1997). Furthermore, the externally studentized residuals were performed to detect potential outliers, whose value higher than 2 would be considered to underlying outliers. To test whether it actually impacted the overall incidence, the influence plot was conducted to identify important outliers, which would be marked with red. The overall analyses were performed with the “metafor” and “meta” packages in R version 3.4.4.

## Results

### Study Characteristics

**Figure 1:**
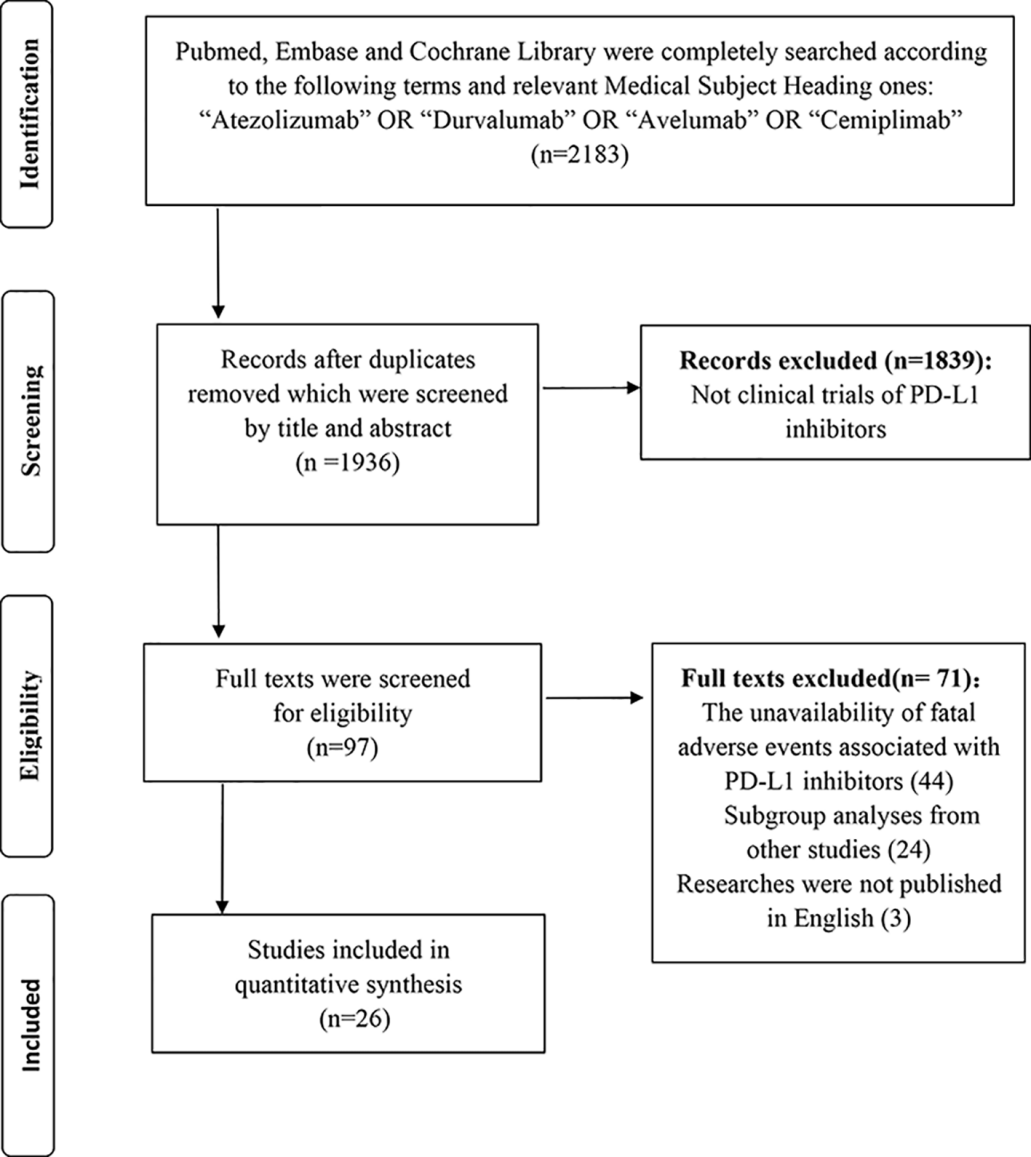
Flow diagram of the meta-analysis.

As **Figure 1** showed, a total of 2,183 relevant records to the PD-L1 inhibitors were retrieved. Thereinto, 1,936 records were screened by the title and abstract after removing the duplicates. Eventually, 26 studies with 6,896 unique individuals were collected following perusing the full texts (Antonia et al., 2016; Fehrenbacher et al., 2016; Balar et al., 2017; Peters et al., 2017; Powles et al., 2017; Barlesi et al., 2018; Choueiri et al., 2018; Dirix et al., 2018; Garassino et al., 2018; Horn et al., 2018; Liu et al., 2018; McDermott et al., 2018; Pal et al., 2018; Patel et al., 2018; Powles et al., 2018; Schmid et al., 2018; Siu et al., 2018; Socinski et al., 2018; Spigel et al., 2018; Chung et al., 2019; Eng et al., 2019; Hong et al., 2019; Kim, 2019; Motzer et al., 2019; Rini et al., 2019; West et al., 2019). In sum, 74 cases of fatal adverse events associated with PD-L1 inhibitors were documented.

Of four PD-L1 inhibitors (atezolizumab, durvalumab, avelumab, cemiplimab), there was no death related to cemiplimab detected. Besides, the most used PD-L1 inhibitor was atezolizumab and the most recorded carcinoma was lung cancer followed by urothelial carcinoma (UC) and renal cell carcinoma (RCC). To detailedly exhibit the difference of incidence among diverse cancers, we totally reported the unique incidence of nine types of cancer. There were two studies with phase I/II involved in durvalumab, which were categorized into phase I study, reporting three treatment-related deaths (Powles et al., 2017; Hong et al., 2019). Furthermore, a study regarding durvalumab exhibited seven treatment-related death, three of which mixed six fatal causes (Kim, 2019). Additionally, two studies included all patients with a positive status of PD-L1 (Peters et al., 2017; Spigel et al., 2018), whereas only one study that PD-L1 status was a negative status (Siu et al., 2018). Each of the following five kinds of cancers only included a qualified article: SCLC, pancreatic cancer, colorectal cancer, gastric or gastroesophageal junction cancer, and head and neck squamous cell carcinoma (HNSCC) (Horn et al., 2018; Siu et al., 2018; Chung et al., 2019; Eng et al., 2019; Hong et al., 2019). A detailed characteristic of 26 studies was presented in **Table 1**. Additionally, the characteristic and the literature list of the ineligible 44 studies were respectively presented in **Supplement Table 1** and **Supplement Table 2**.

**Table 1:** Characteristics of eligible studies.

**Study Characteristics**		**Eligible studies** **(n = 26)**	**Number of patients**
**PD-L1 inhibitors**	Atezolizumab	14	4,053
	Durvalumab	6	1,394
	Avelumab	6	1,449
**Cancer type**	NSCLC	8	2,428
	Urothelial carcinoma	5	1,232
	Renal cell carcinoma	4	1,041
	Breast cancer	2	620
	Non-squamous NSCLC	2	866
	SCLC	1	198
	Pancreatic cancer	1	49
	Colorectal cancer	1	179
	Gastric or gastroesophageal junction cancer	1	150
	HNSCC	1	133
**Trial phase**	III	10	3,907
	II	8	1,949
	I*	8	1,040
**PD-L1 status**	Mixed^	23	5,967
	Positive	2	796
	Negative	1	133
**Race**	Mixed^$^	18	5,930
	Non-East Asia^#^	8	966
**Gender**	Mixed^##^	24	6,276
	Female	2	620
**Treatment line**	Prior treatment	17	4,220
	First-line	9	2,676
**Average age**	≤62	11	2,657
	<67	10	3,007
	≥67	5	1,232

PD-L1, programmed cell death ligand 1; NSCLC, non-small cell lung cancer; SCLC, small cell lung cancer; HNSCC, head and neck squamous cell carcinoma.

*Two studies with phase I/Ⅱ were categorized into phase I study.

^PD-L1 status of different patients in these study included the following three conditions: positive, negative, and/or unknown.

^$^Participants in these study included East Asians and non-East Asians.

^#^Participants in these study did not include Chinese, Japanese, Korean.

^##^Participants in these study included male and female.

### Incidence of Fatal Adverse Events Associated With PD-L1 Inhibitors

**Figure 2:**
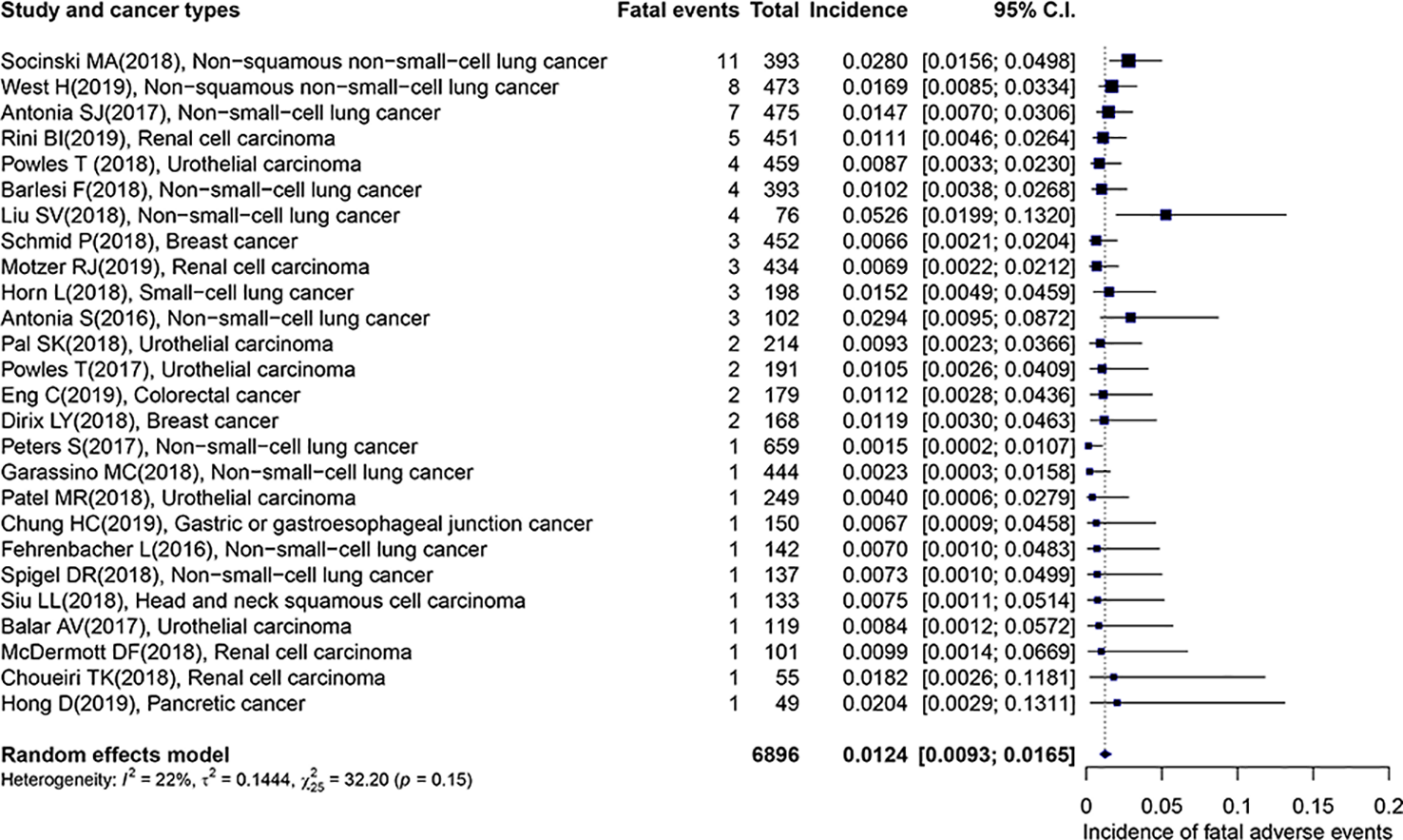
Forest plot of the overall incidence of fatal adverse events associated with PD-L1 inhibitors.

The overall incidence of fatal adverse events associated with PD-L1 inhibitors was 1.24% (95% CI: 0.93–1.65%), which displayed in **Figure 2**. Importantly, the heterogeneity was not detected in this study (*I* ^2^ = 22%, *p* = 0.15). The subgroup analyses were performed based on the following factors: the kind of PD-L1 inhibitors, the type of cancer, the trial phase of studies, the PD-L1 status, the race, the gender, the treatment line, and the average age of patients. The detailed results were presented in **Table 2**. Of note, the incidence of patients with non-squamous NSCLC was evidently higher than those with UC (2.25 vs. 0.85, *p =* 0.04). In comparison with the young group, patients from the middle-aged group possessed a significantly higher incidence (1.74 vs. 0.89, *p =* 0.01). Compared with trial phase II, the incidence of trial phase I was markedly higher (1.76 vs. 0.60, *p =* 0.01). As for the analysis of PD-L1 status, we observed that the incidence of patients with positive PD-L1 status was less than half that in patients with negative PD-L1 status (0.33 vs. 0.75, *p =* 0.53). In addition, the patients with PD-L1 positive expression hold a notable lower incidence compared to those with mixed PD-L1 status (0.33 vs. 1.33, *p =* 0.07). In terms of the type of PD-L1 inhibitors, the incidence of atezolizumab (1.29 vs. 0.89, *p =* 0.27) and durvalumab (1.39 vs. 0.89, *p =* 0.38) was greater than that of avelumab. Given non-squamous NSCLC had a markedly higher incidence, the difference across three types of lung cancer was detected. In comparison with NSCLC, there was no significant difference in the incidence of non-squamous NSCLC (2.25 vs. 1.13, *p =* 0.25) or SCLC (1.52 vs. 1.13, *p =* 0.89).

**Table 2:** Results of subgroup analyses and univariate meta-regression of fatal adverse events associated with PD-L1 inhibitors.

Fatal adverse events					
Subgroup	Total studies	Incidence (%) (95% CI)	OR (95% CI)	P
**Overall**	26	1.24 (0.93–1.65)	NA	NA
**PD-L1 inhibitors**	Durvalumab	1.39 (0.84–2.30)	1.51 (0.61–3.75)	0.38
	Atezolizumab	1.29 (0.85–1.95)	1.53 (0.72–3.27)	0.27
	Avelumab	0.89 (0.51–1.56)	REF	
**Cancer type**	Non-squamous NSCLC	2.25 (1.37–3.66)	2.69 (1.04–6.97)	0.04*
	Pancreatic cancer	2.04 (0.29–13.11)	2.47 (0.27–22.20)	0.42
	SCLC	1.52 (0.49–4.59)	1.82 (0.41–8.04)	0.43
	NSCLC	1.13 (0.51–2.47)	1.66 (0.70–3.92)	0.25
	Colorectal cancer	1.12 (0.28–4.36)	1.34 (0.25–7.23)	0.74
	Renal cell carcinoma	1.00 (0.54–1.85)	1.20 (0.44–3.29)	0.72
	Breast cancer	0.84 (0.35–2.00)	1.01 (0.30–3.42)	0.98
	HNSCC	0.75 (0.11–5.14)	0.90 (0.10–7.98)	0.92
	Gastric or gastroesophageal junction cancer	0.67 (0.09–4.58)	0.79 (0.09–7.06)	0.84
	Urothelial carcinoma	0.85 (0.46–1.56)	REF		
**Trial phase**	III	1.33 (0.96–1.85)	0.70 (0.36–1.35)	0.29
	II	0.60 (0.31–1.14)	0.31 (0.13–0.75)	0.01*
	I	1.76 (0.93–3.30)	REF		
**Race**	Non-East Asia^#^	1.79 (0.96–3.29)	1.69 (0.86-3.31)	0.13
	Mixed^$^	1.11 (0.81–1.52)	REF		
**PD-L1 status**	Negative	0.75 (0.11–5.14)	2.27 (0.18–28.91)	0.53
	Mixed^	1.33 (1.00–1.77)	4.04 (0.91–18.02)	0.07
	Positive	0.33 (0.07–1.54)	REF		
**Gender**	Female	0.84 (0.30–2.00)	0.66 (0.23–1.93)	0.45
	Mixed^#^^#^	1.27 (0.94–1.92)	REF		
**Treatment line**	First-line	1.39 (0.91–2.11)	1.20 (0.66–2.17)	0.55
	Prior treatment	1.12 (0.75–1.68)	REF		
**Average age**	≥67	0.85 (0.46–1.56)	0.95 (0.45–2.03)	0.90
	< 67	1.74 (1.15–2.63)	2.13 (1.26–3.61)	0.01*
	≤62	0.89 (0.58–1.36)	REF		

CI, confidence interval; OR, odds ratio; NA, not available; PD-L1, programmed cell death ligand 1; REF, reference group; NSCLC, non-small cell lung cancer; SCLC, small cell lung cancer; HNSCC, head and neck squamous cell carcinoma.

*A value of P < 0.05 indicated statistical significance.

^#^Participants in these study did not include Chinese, Japanese, and Korean.

^$^Participants in these study included East Asians and non-East Asians.

^PD-L1 status of different patients in these study included the following three conditions: positive, negative, and unknown.

^##^Participants in these study included male and female.

As for the univariate meta-regression analysis, the results demonstrated that the odds of patients with non-squamous NSCLC was evidently higher than those with UC (odds ratio [OR]: 2.69; 95% CI: 1.04–6.97, *p =* 0.04). In comparison with the young group, patients from the middle-aged group possessed significantly higher odds (OR: 2.13; 95% CI: 1.26–3.61, *p* = 0.01). Compared with trial phase I, the odds of trial phase II were markedly lower (OR: 0.31; 95% CI: 0.13–0.75, *p =* 0.01). There was no marked difference for the odds across the type of PD-L1 inhibitors, the gender, the treatment line, the race, and the PD-L1 status.

Considering the results of univariate meta-regression analysis, the needs of clinical practice and sample size of our study, we merely included the following factors for multivariate meta-regression analysis: cancer types, the average age of patients, and the trial phase of the study. The detailed results were presented in **Table 3**. Notably, the patients who were enrolled in trial phase I had a markedly higher incidence than those in phase II or phase III following regulating for cancer types and average age (OR: 0.28; 95% CI: 0.11–0.71, *p* = 0.01, OR: 0.48; 95% CI: 0.24–0.95, *p* = 0.04, respectively). However, there was no significant discrepancy between non-squamous NSCLC and UC following adjusting for the average age and trial phase. Also, an analogous trend was observed in different age groups after controlling for average age and cancer types.

**Table 3:** Results of multivariate meta-regression of fatal adverse events associated with PD-L1 inhibitors.

**Fatal adverse events**			
Variables		OR (95% CI)	P
**Cancer type**	Non-squamous NSCLC	0.91 (0.08–10.44)	0.94
	Pancreatic cancer	0.78 (0.04–14.64)	0.87
	SCLC	0.61 (0.04–8.60)	0.71
	NSCLC	0.55 (0.05–5.64)	0.61
	Colorectal cancer	0.88 (0.07–11.32)	0.92
	Renal cell carcinoma	0.72 (0.08–6.42)	0.77
	Breast cancer	0.49 (0.05–4.89)	0.54
	Gastric or gastroesophageal Junction cancer	0.25 (0.01–4.67)	0.35
	HNSCC^#^	NA^#^	NA
	Urothelial carcinoma	REF	
**Trial phase**	III	0.48 (0.24–0.95)	0.04*
	II	0.28 (0.11–0.71)	0.01*
	I	REF	
**Average age**	≥67	0.62 (0.07–5.29)	0.67
	< 67	1.98 (0.59–6.67)	0.27
	≤62	REF	

OR, odds ratio; NSCLC, non-small cell lung cancer; SCLC, small cell lung cancer; HNSCC, head and neck squamous cell carcinoma; NA, not available; REF, reference group;

*A value of P < 0.05 indicated statistical significance.

^#^Redundant predictor was dropped from the analysis.

### The Evaluation of Potential Outliers

**Figure 3:**
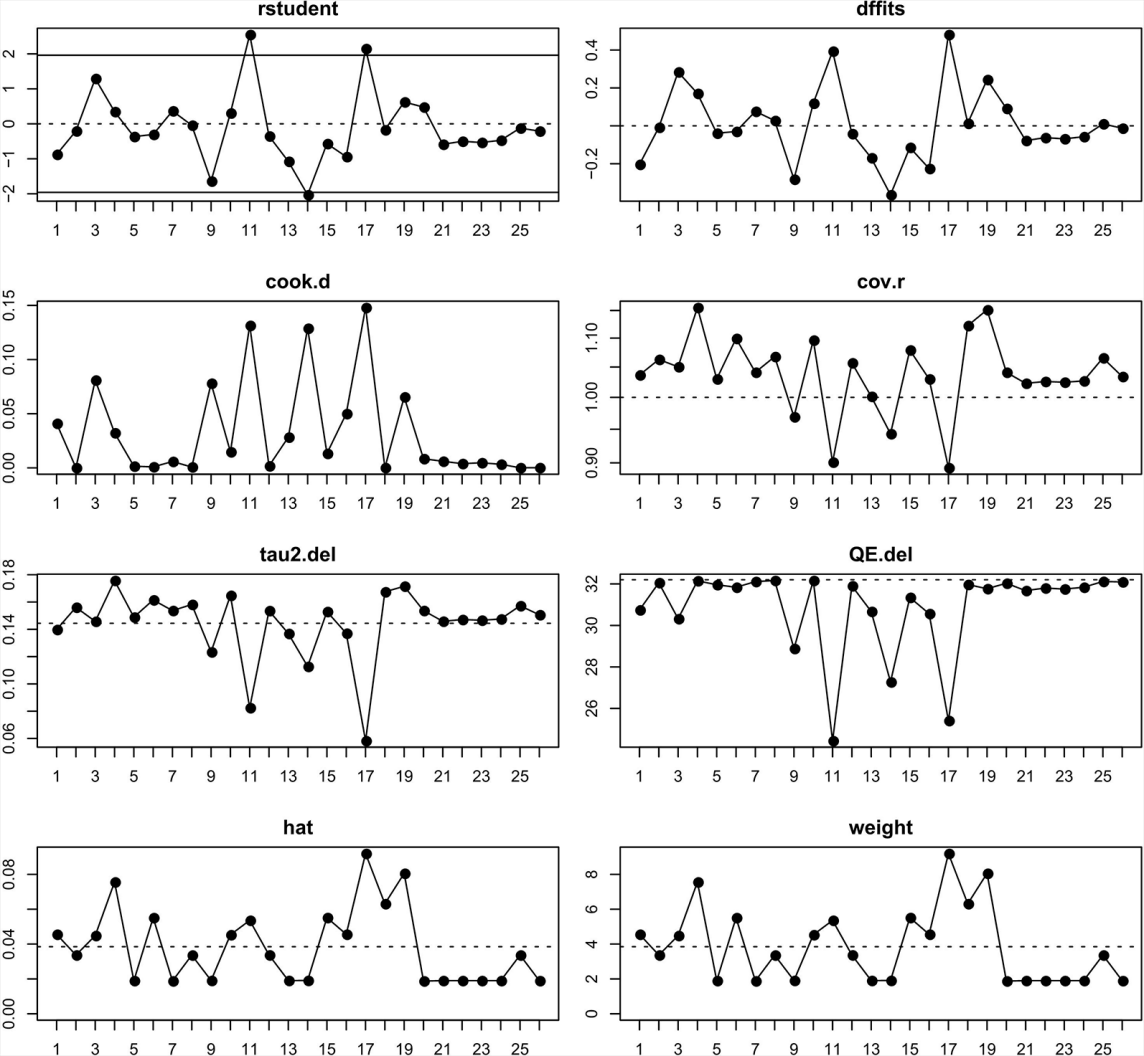
The plot of influential study. The influential studies would be marked with red.

From **Figure 2** we noted that some underlying outliers might exist in the study and impacted the overall incidence. Hence, the externally studentized residuals were performed to detect potential outliers. The results demonstrated that there were three studies had a value of z higher than 2 (Peters et al., 2017; Liu et al., 2018; Socinski et al., 2018) (z = 2.1, 2.5, 2.0, respectively). To test whether these studies actually impacted the overall incidence, the influence plot was conducted to identify important outliers. The results exhibited that the outliers did not exist in our study (**Figure 3**). In addition, the details of the 26 eligible studies shown in **Figure 3** which were presented in **Supplement Table 3**.

### The Spectrum of Fatal Adverse Events Associated With PD-L1 Inhibitors

To provide a detailed spectrum of fatal adverse events associated with PD-L1 inhibitors, we drastically reviewed the causes of death across 74 fatal cases. A total of 40 fatal causes were recorded. Intriguingly, ILD was the leading cause (n = 12), ensuing major fatal causes were sepsis (n = 5), neutropenia (n = 4), pneumonia (n = 3), hemoptysis (n = 3), respiratory failure (n = 3), autoimmune hepatitis(n = 3), myocarditis (n = 2), pulmonary hemorrhage (n = 2), intestinal obstruction (n = 2), intracranial hemorrhage (n = 2), septic shock (n = 2), sudden death (n = 2). The complete spectrum was presented in **Table 4**. Further analysis based on organ-specific fatal adverse events associated with PD-L1 inhibitors, we found that the respiratory system (n = 24) was the most frequently reported, followed by the digestive system (n = 9), the circulatory system (n = 9), the nervous system (n = 6), and the urinary system (n = 2). Additionally, other results were integrated based on following organ-unspecific fatal adverse events: the inflammatory infection (n = 12), the cutaneous adverse events (n = 2), and others (n = 4). What needed to be emphasized was that three patients occurred multiorgan-specific fatal adverse events, comprising autoimmune myocarditis, acute cardiac failure, and respiratory failure (Barlesi et al., 2018); multiple organ dysfunction syndromes (MODS) (Rini et al., 2019); autoimmune hepatitis and hepatic failure (Chung et al., 2019), respectively. Besides, we also noted that the causes of death among the other three patients were a mixture of the following six conditions: cardiomyopathy, right ventricular failure, respiratory distress, respiratory failure, brain natriuretic peptide increased, and radiation pneumonitis (Kim, 2019). In consideration of the special cases mentioned above, we presented the results of the six patients as the separate group in **Table 4**. Additionally, all cases of myocarditis were reported in the avelumab group (Choueiri et al., 2018; Motzer et al., 2019).

**Table 4:** The spectrum of fatal adverse events associated with PD-L1 inhibitors.

	The cause of death	No. (%)
The respiratory system	interstitial lung disease	12(16.22)
	pneumonia	3(4.05)
	respiratory failure	3(4.05)
	hemoptysis	3(4.05)
	pulmonary hemorrhage	2(2.70)
	respiratory distress	1(1.35)
Total		**24(32.42)**
The digestive system	autoimmune hepatitis	3(4.05)
	intestinal obstruction	2(2.70)
	necrotizing pancreatitis	1(1.35)
	acute liver failure	1(1.35)
	intestinal perforation	1(1.35)
	hepatic cirrhosis	1(1.35)
Total		**9(12.15)**
The circulatory system	myocarditis	2(2.70)
	pericardial effusion	1(1.35)
	aortic dissection	1(1.35)
	myocardial infarction	1(1.35)
	cardiac arrest	1(1.35)
	ventricular tachycardia	1(1.35)
	constrictive pericarditis	1(1.35)
	cardiac failure	1(1.35)
Total		**9(12.15)**
The nervous system	intracranial hemorrhage	2(2.70)
	myasthenia gravis	1(1.35)
	neuromuscular disorder	1(1.35)
	cerebrovascular accident	1(1.35)
	cerebral infarction	1(1.35)
Total		**6(8.10)**
The urinary system	adrenal insufficiency	1(1.35)
	acute kidney injury	1(1.35)
Total		**2(2.70)**
The inflammatory infection	sepsis	5(6.76)
	neutropenia	4(5.41)
	septic shock	2(2.70)
	systemic candida infection	1(1.35)
Total		**12(16.22)**
The cutaneous events	toxic epidermal necrolysis	1(1.35)
	mucosal inflammation	1(1.35)
Total		**2(2.70)**
The multiorgan-specific	autoimmune hepatitis and hepatic failure	1(1.35)
	autoimmune myocarditis, acute cardiac failure, and respiratory failure	1(1.35)
	multiple organ dysfunction syndrome	1(1.35)
Total		**3(4.05)**
Mixed^#^	Cardiomyopathy, right ventricular failure, respiratory distress, respiratory failure, brain natriuretic peptide increased, radiation pneumonitis	3(4.05)
Total		**3(4.05)**
Others	sudden death	2(2.70)
	General physical health deterioration	1(1.35)
	unknown cause	1(1.35)
Total		**4(5.40)**
Total		74(99.94*)

^#^the causes of death among the unique three patients were a mixture of the following six conditions: cardiomyopathy, right ventricular failure, respiratory distress, respiratory failure, brain natriuretic peptide increased, and radiation pneumonitis.

*The percentages do not add up to 100% due to rounding.

### Publication Bias

**Figure 4:**
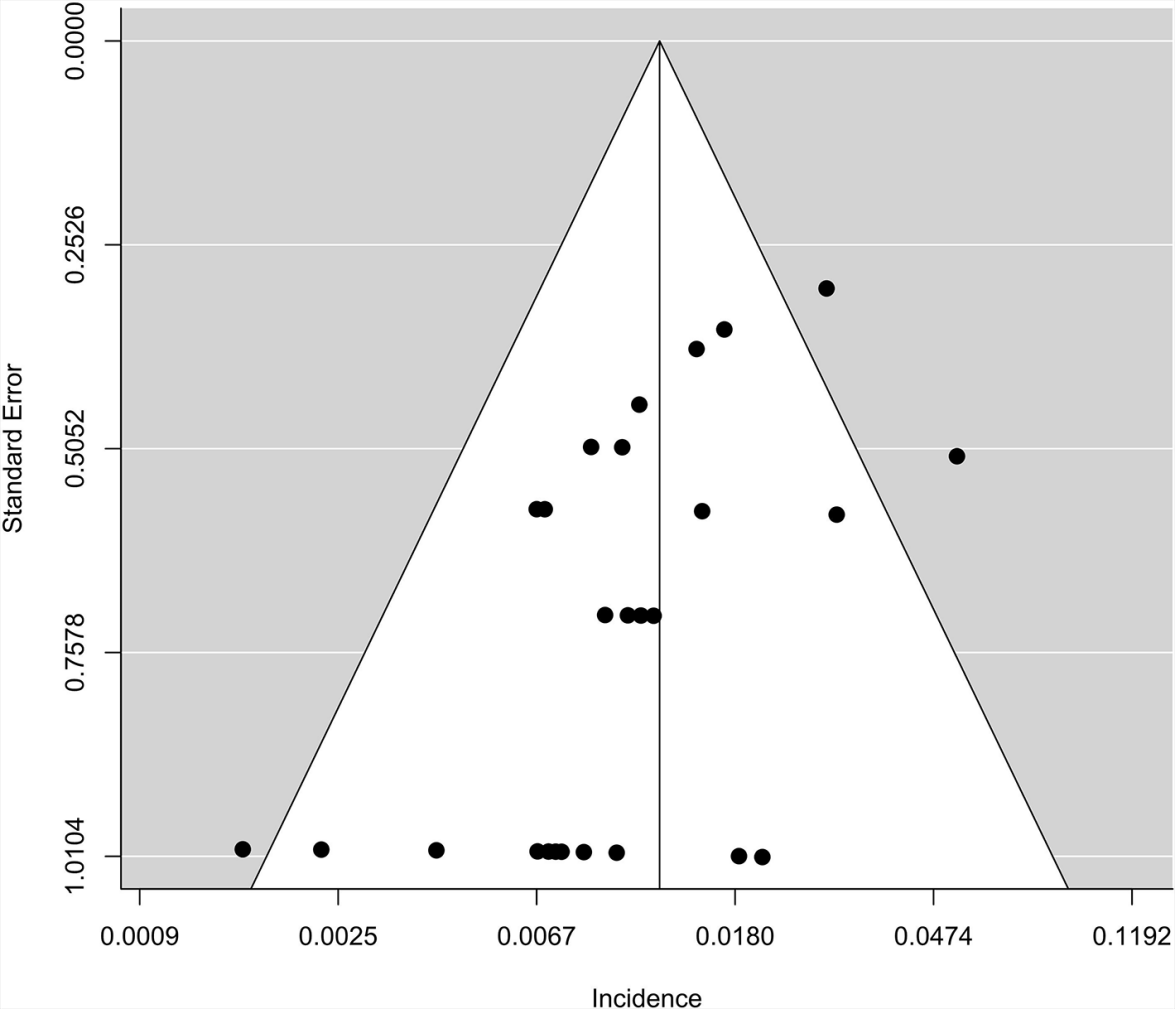
The funnel plot of publication bias in the meta-analysis.

The funnel plot regarding the incidence of fatal adverse events associated with PD-L1 inhibitors was asymmetric (**Figure 4**). A publication bias existed in this study based on Egger test (p < 0.01).

## Discussion

Despite promising progress in the treatment regimen of anti-cancer represented by PD-L1 inhibitors, treatment-related death occasionally emerged. The safety of PD-L1 inhibitors was an essential field to profoundly explore. Therefore, we conducted a meta-analysis involving 26 studies with 6,896 unique participants to elucidate the incidence, the detailed spectrum, and the potential susceptible factors of fatal adverse events associated with PD-L1 inhibitors. The results demonstrated that the overall incidence was 1.24% (95% CI: 0.93–1.65%). In terms of the analysis of spectrum, we observed that ILD was the leading cause of death (n = 12) followed by sepsis (n = 5), neutropenia (n = 4), pneumonia (n = 3), hemoptysis (n = 3), respiratory failure (n = 3), and autoimmune hepatitis (n = 3). Importantly, two rare deaths from PD-L1 inhibitors, relating to myocarditis were documented. As for the analysis of the organs-specific fatal adverse events, the respiratory system (n = 24) was the most frequently reported. According to the results of univariate meta-regression analysis, the potential susceptible factors were as below: the patients treated with PD-L1 inhibitors who involved non-squamous NSCLC, who were included in the trial phase I, and who from the middle-aged group. Additionally, the trial phase of the study was a confirmative predictor based on the results of multivariate meta-regression analysis. Importantly, the aforementioned results, including the incidence, the complete spectrum, the potential susceptible factors, and the relevant predictors of the fatal adverse events associated with PD-L1 inhibitors were firstly reported *via* searching widespread databases.

There was a meta-analysis displayed the incidence of general adverse events related to PD-1 and PD-L1 inhibitors (Baxi et al., 2018), including fatigue, rash, hepatitis, pneumonitis, and diarrhea. However, our study concentrated on fatal adverse events instead of general adverse events. In addition, our study exhibited the incidence of the fatal adverse events associated with PD-L1 inhibitors was 1.24%, which was remarkably distinct from those reported from the previous meta-analysis incorporated one clinical trial regarding atezolizumab, whose incidence was 0.63% (Abdel-Rahman et al., 2017). The evident distinction might origin from significant different sample sizes (n = 26 vs. n = 1). Also, our results were obviously distinct from the prior study that reported the real-world incidence was 1.97% (613 unique fatal irAEs were identified from 31,059 irAEs case reports) according to the global adverse drug reaction database (Vigilyze-Vigibase) and the pooled incidence was 0.8% based on the pooled meta-analysis (Wang et al., 2018). The distinction might be attributed to different inclusion criteria and sample sizes (n = 26 vs. n = 15). Intriguingly, When the ineligible 44 studies with zero fatal events were added to analyze, the re-evaluated overall incidence slightly altered (1.15%, 95% CI: 0.91–1.45%).

This study provided the important implications for clinical safety usage of PD-L1 inhibitors. The results revealed that old patients had a higher incidence of fatal adverse events than young patients. The reasonable interpretation of that was the old patients frequently complicated with a series of basic diseases. Consequently, the old patients were more likely to be threatened by fatal adverse events associated with PD-L1 inhibitors, which implied that the clinicians need to monitor old patients more closely when receiving PD-L1 inhibitors.

Moreover, the results also demonstrated that the incidence of patients with non-squamous NSCLC was evidently higher than those with UC. The potential rationale of that might be attributed to the following two factors. One possibility was that a great distinction of PD-L1 expression between NSCLC and UC. Compared with the latter, the former had a remarkable expression of PD-L1 (Patel and Kurzrock, 2015). Besides, partial studies regarding patients with NSCLC or UC revealed that the high levels of PD-L1 expression were related to favorable overall response rate (ORR) and OS (Velcheti et al., 2014; Bellmunt et al., 2015; Brody et al., 2017; Ghate et al., 2019). Further studies exhibited that irAEs were significantly associated with the efficacy of ICIs in patients with NSCLC or melanoma (Hua et al., 2016; Haratani et al., 2018). The above studies seemingly indicated that the PD-L1 expression was positively associated with irAEs. However, we also observed a paradoxical phenomenon that appeared in this study. The incidence in the PD-L1-positive expression group was numerically lower than those in PD-L1-negative expression (0.33 vs. 0.75, *p =* 0.53). Additionally, a marginally significant difference was discovered in the PD-L1-positive group and mixed ones (0.33 vs. 1.33, *p =* 0.07). Based on the above contradictory shreds of evidence, further researches were needed to carry out to elucidate the relationship between them. Despite the ambiguity, this study provided a preliminary insight that the therapy of PD-L1 inhibitors in patients with UC was safer than those with non-squamous NSCLC, which indicated that the clinicians should particularly monitor the adverse events occurred in patients with non-squamous NSCLC in case of the emergent of fatal adverse events. Alternatively, the different tumor burden existed in non-squamous NSCLC and UC. The prior study suggested that a high tumor burden was related to serious irAEs in patients with NSCLC (Sakata et al., 2019), which forcefully implied that tumor burden was likely to be an underlying predictor for fatal adverse events associated with PD-L1 inhibitors. There is abundant room for further progress in determining the correlation between them.

The incidence of fatal adverse events associated with PD-L1 inhibitors was higher in phase I than that in phase II according to univariate meta-regression analysis. Intriguingly, after regulating for cancer types and average age, the incidence of phase I had a markedly higher incidence than that in phase II or phase III. The reasonable interpretation was that the design of subsequent trials was optimized based on the experience of early trials to promote the reduction of incidence.

To our knowledge, the predictors of fatal adverse events remained unclear. There were some studies indicated that the occurrence of fatal events might be related to unique genes or specific gastrointestinal flora (Wolchok et al., 2010; Chaput et al., 2017). In this study a total of three potential predisposing factors (non-squamous NSCLC, the middle-aged group, and the trial phase I) based on the results of univariate meta-regression analysis, whereas only a relevant predictor (the trial phase I) was established according to multivariate meta-regression analysis. These easily available clinical characteristics could promote clinicians to quickly predict and identify high-risk populations, and facilitate clinicians to early detect and effectively reduce these thorny issues occurred.

Of note, the amount of respiratory system-related fatal adverse events (n = 24, 32.43%) were predominate across various fatal causes, with ILD being the most prominent (n = 12, 16.22%). ILD, mainly including idiopathic pulmonary fibrosis and pneumonitis in our study, is a group that incorporates more than 200 diseases characterized by diffuse pulmonary parenchymal disease. Beyond that, the increased awareness of the sharp development of circulatory system-related adverse events (e.g. myocarditis), the improved knowledge of extremely rare neurological-related ones (e.g. myasthenia gravis), and the enhanced comprehension of inflammatory infection-related ones (e.g. sepsis and neutropenia) that can be completely reversed by early intervention would develop the safety of using PD-L1 inhibitors in the clinical setting.

PD-L1 inhibitors have shown remarkable antitumor benefit by improving the activity of the immune system *via* blocking the binding of PD-1 to PD-L1. Unfortunately, however, excessive enhancement of immune system activity could trigger inflammatory side effects, which was termed irAEs. There was some evidence that might explain the underlying mechanism of fatal irAEs. Firstly, homologous antigens existed in some tumors and normal tissues. A report with two fatal myocarditis exhibited that similar T-cell clones were observed in both the tumor and the myocardium (Johnson et al., 2016). Hence the myocardium would be attacked by activated T cells, and developed cardiac-related irAEs. Secondly, IgG-dependent complement fixation and phagocytosis, which would over-activate the immune system, triggering extensive destruction on normal tissue with high CTLA-4 expression (Caturegli et al., 2016) (e.g. pituitary gland). Besides that, the release of substantial cytokines might be involved in the occurrence of irAEs, of which interleukin-17 (IL-17) was the best known. Multiple studies implied that the elevated level of IL-17 was associated with immune-related colitis (Samaan et al., 2018). Additionally, beyond T-cell-mediated immunity, anti-PD-L1 therapy could modulate humoral immunity by increasing levels of preexisting antibodies (Osorio et al., 2017) (e.g. antithyroid antibodies). Despite the precise mechanism, irAEs were attributed to excessive immunity against normal tissue or organs (Postow et al., 2018).

There were several limitations existed in this study. Firstly, this pooled analysis was conducted by using aggregated patient data instead of individual data, which limited the capability to deeply explore the correlation between adverse events and characteristics of patients. Additionally, given the absence of the control group in some studies, the comparison of PD-L1 inhibitors with other drugs could not be performed. Consequently, the pooled analysis merely involved the difference in the incidence of fatal adverse events in diverse PD-L1 inhibitors. Furthermore, given the number of studies regarding PD-L1-positive expression extremely little (n = 2), the role of PD-L1 expression in the incidence of fatal adverse events associated with PD-L1 inhibitors remained controversial.

## Conclusion

The overall incidence of fatal adverse events associated with PD-L1 inhibitors was 1.24%, which was markedly higher in patients involved non-squamous NSCLC, trial phase I, and the middle-aged group, compared with the corresponding reference group. The amounts of respiratory system-related fatal adverse events predominated across various fatal causes, of that ILD being the most prominent.

This study firstly provided three potential susceptible factors (non-squamous NSCLC, trial phase I, and the middle-aged group) and ultimately established a relevant predictor (trial phase I) according to multivariate meta-regression analysis, which yielded a capability for clinicians to distinguish high-risk populations from relatively low-risk ones, facilitating to improve the safety of PD-L1 inhibitors which were broadly used in the clinical setting. Importantly, given the maturity of the PD-L1 expression detection methods, we urgently hoped that a variety of studies regarding the correlation between PD-L1 expression and fatal adverse events occurred, prompting clinicians to early detect and effectively handle these thorny issues.

## Data Availability Statement

All datasets generated for this study are included in the article/**Supplementary Material**.

## Author Contributions

XW: project design, data analysis, manuscript writing. SW: draft the article, data analysis. YC, ES, TZ, and LL: data collection, data extraction. XC: critically edited the manuscript, supervised the project.

## Funding

This research received financial support from the National Natural Science Foundation of China (Nos 81502360); the Natural Science Foundation of Fujian Province (Nos 2018J01352, 2016J01576, and 2016J01586); the Science and Technology Innovation Joint Foundation of Fujian Province(Nos 2017Y9125).

## Conflict of Interest

The authors declare that the research was conducted in the absence of any commercial or financial relationships that could be construed as a potential conflict of interest.
