# Correction: Fatal adverse events associated with programmed cell death ligand 1 inhibitors: a systematic review and meta-analysis

**DOI:** 10.3389/fphar.2026.1782058

**Published:** 2026-02-17

**Authors:** Xuewen Wang, Shijie Wu, Yaying Chen, Erqian Shao, Tingting Zhuang, Linbin Lu, Xiong Chen

**Affiliations:** 1 Department of Oncology, The 900th Hospital of the People’s Liberation Army Joint Service Support Force, Fuzong Clinical Medical College of Fujian Medical University, Fuzhou, China; 2 Department of Clinical Medicine, Fujian Medical University, Fuzhou, China

**Keywords:** PD-L1 inhibitors, fatal adverse events, incidence, meta-analysis, interstitial lung disease

A correction has been made to section **Methods**, *Statistical Methods*. This sentence previously stated:

“The funnel plot and Egger or Begger’s tests were used to estimate the publication bias of this study (**Egger et al., 1997**).”

The corrected sentence appears below:

“The funnel plot and Egger tests were used to estimate the publication bias of this study (**Egger et al., 1997**).”

A correction has been made to section **Results**, *Publication Bias*. This sentence previously stated:

“However, there was no evidence to prove publication bias existed in this study based on Begger’s test (*p* = 0.57). Moreover, when 26 eligible studies and 44 ineligible ones were included in the analysis, there was a significant publication bias based on Begger’s test (*p* < 0.0001).”

The corrected sentence appears below:

“A publication bias existed in this study based on Egger test (p < 0.01).”

A correction has been made to section **Discussion**, Paragraph 2. The final sentence of the paragraph, appearing below, was removed:

“However, the inclusion of these studies would introduce substantial bias based on the Begger’s test (p < 0.0001).”

The original article has been updated.

