# Supplementary material for: Fatal Adverse Events Associated With Programmed Cell Death Ligand 1 Inhibitors: A Systematic Review and Meta-Analysis
Source: Front Pharmacol. 2020 Jan 31;11:5. doi: 10.3389/fphar.2020.00005 (PMC7006642; doi:10.3389/fphar.2020.00005)
Supplement: Supplementary file 1 [file Table_1.doc]

**Supplement table 1. Characteristics of the 44 ineligible studies**

| **Author year** | **Cancer type** | **PD-L1 inhibitors** | **Fatal adverse events** | **Total** |
| --- | --- | --- | --- | --- |
| Petrylak DP (2018) | Urothelial cancer | Atezolizumab | 0 | 95 |
| Rosenberg JE (2016) | Urothelial cancer | Atezolizumab | 0 | 310 |
| Necchi A (2017) | Urothelial cancer | Atezolizumab | 0 | 220 |
| Sullivan RJ (2019) | Melanoma | Atezolizumab | 0 | 57 |
| Gandara DR (2018) | Non-small cell lung cancer | Atezolizumab | 0 | 425 |
| Rittmeyer A (2017) | Non-small cell lung cancer | Atezolizumab | 0 | 609 |
| McDermott DF (2016) | Renal cell carcinoma | Atezolizumab | 0 | 70 |
| Heery CR (2017) | Solid tumor | Avelumab | 0 | 53 |
| Gulley JL (2017) | Non-smal cell lung cancer | Avelumab | 0 | 184 |
| Kaufman HL (2016) | Merkel cell carcinoma | Avelumab | 0 | 88 |
| Le Tourneau (2018) | Adrenocortical carcinoma | Avelumab | 0 | 50 |
| Apolo AB (2017) | Urothelial cancer | Avelumab | 0 | 44 |
| Lukas RV (2018) | Glioblastoma | Atezolizumab | 0 | 16 |
| Levy A (2016) | Solid tumor | Durvalumab | 0 | 10 |
| Rebelatto MC (2016) | Non-small cell lung cancer and head and neck squamous cell carcinoma | Durvalumab | 0 | 181 |
| Paz-Ares L (2019) | Small cell lung cancer | Durvalumab | 0 | 268 |
| Rotman J (2018) | Cervical cancer | Durvalumab | 0 | 9 |
| D'Angelo SP (2019) | Merkel cell carcinoma | Avelumab | 0 | 88 |
| D'Angelo SP (2018) | Merkel cell carcinoma | Avelumab | 0 | 39 |
| Rajan A (2019) | Thymoma/Thymic Carcinoma | Avelumab | 0 | 8 |
| Mignard X (2018) | Non-small cell lung cancer | Durvalumab | 0 | 81 |
| Yu Y (2019) | Head and neck cancer | Avelumab | 0 | 320 |
| Moehler M (2019) | Gastric cancer | Avelumab | 0 | 250 |
| Necchi A (2019) | Germ cell tumors | Durvalumab | 0 | 22 |
| Chih-Hsin Yang J (2019) | Non-small cell lung cancer | Durvalumab | 0 | 12 |
| Migden MR (2018) | Cutaneous squamous-cell carcinoma | Cemiplimab | 0 | 26 |
| Doi T (2019) | Advanced solid tumors | Avelumab | 0 | 17 |
| Mizugaki H (2016) | Solid tumors | Atezolizumab | 0 | 6 |
| Bahig H (2019) | Head and neck cancer | Durvalumab | 0 | 12 |
| Chia S (2019) | Breast cancer | Durvalumab | 0 | 15 |
| Mego M (2019) | Germ cell cancer | Avelumab | 0 | 8 |
| Bang YJ (2018) | Gastric or gastroesophageal junction cancer | Avelumab | 0 | 59 |
| Shemesh CS (2019) | Solid tumors | Atezolizumab | 0 | 87 |
| Loibl S (2019) | Breast cancer | Durvalumab | 0 | 174 |
| Pujol JL (2019) | Small cell lung cancer | Atezolizumab | 0 | 49 |
| Colevas AD (2018) | Head and neck cancer | Atezolizumab | 0 | 32 |
| Horn L (2018) | Non-small cell lung cancer | Atezolizumab | 0 | 89 |
| Allen CT (2019) | Respiratory papillomatosis | Avelumab | 0 | 12 |
| Lee JM (2017) | Solid tumors | Durvalumab | 0 | 26 |
| Massard C (2016) | Urothelial bladder cancer | Durvalumab | 0 | 61 |
| Liu JF (2019) | Ovarian cancers/ Uterine cancer | Atezolizumab | 0 | 27 |
| Fujiwara Y (2019) | Advanced solid tumors | Durvalumab | 0 | 22 |
| Calabro L (2018) | Mesothelioma | Durvalumab | 0 | 40 |
| Kaufman HL (2018) | Merkel cell carcinoma | Avelumab | 0 | 88 |
