# Supplementary material for: Fatal Adverse Events Associated With Programmed Cell Death Ligand 1 Inhibitors: A Systematic Review and Meta-Analysis
Source: Front Pharmacol. 2020 Jan 31;11:5. doi: 10.3389/fphar.2020.00005 (PMC7006642; doi:10.3389/fphar.2020.00005)
Supplement: Supplementary file 2 [file Table_2.doc]

**Supplement table 2. Reference list of the 44 ineligible studies**

1. Petrylak DP, Powles T, Bellmunt J, Braiteh F, Loriot Y, Morales-Barrera R, et al. Atezolizumab (MPDL3280A) Monotherapy for Patients With Metastatic Urothelial Cancer: Long-term Outcomes From a Phase 1 Study. *JAMA oncology* (2018) 4(4):537-44. Epub 2018/02/10. doi: 10.1001/jamaoncol.2017.5440. PubMed PMID: 29423515; PubMed Central PMCID: PMCPMC5885219.

2. Rosenberg JE, Hoffman-Censits J, Powles T, van der Heijden MS, Balar AV, Necchi A, et al. Atezolizumab in patients with locally advanced and metastatic urothelial carcinoma who have progressed following treatment with platinum-based chemotherapy: a single-arm, multicentre, phase 2 trial. *Lancet (London, England)* (2016) 387(10031):1909-20. Epub 2016/03/10. doi: 10.1016/s0140-6736(16)00561-4. PubMed PMID: 26952546; PubMed Central PMCID: PMCPMC5480242.

3. Necchi A, Joseph RW, Loriot Y, Hoffman-Censits J, Perez-Gracia JL, Petrylak DP, et al. Atezolizumab in platinum-treated locally advanced or metastatic urothelial carcinoma: post-progression outcomes from the phase II IMvigor210 study. *Annals of oncology : official journal of the European Society for Medical Oncology* (2017) 28(12):3044-50. Epub 2017/09/28. doi: 10.1093/annonc/mdx518. PubMed PMID: 28950298; PubMed Central PMCID: PMCPMC5834063.

4. Sullivan RJ, Hamid O, Gonzalez R, Infante JR, Patel MR, Hodi FS, et al. Atezolizumab plus cobimetinib and vemurafenib in BRAF-mutated melanoma patients. *Nat Med* (2019) 25(6):929-35. Epub 2019/06/07. doi: 10.1038/s41591-019-0474-7. PubMed PMID: 31171876.

5. Gandara DR, von Pawel J, Mazieres J, Sullivan R, Helland A, Han JY, et al. Atezolizumab Treatment Beyond Progression in Advanced NSCLC: Results From the Randomized, Phase III OAK Study. *Journal of thoracic oncology : official publication of the International Association for the Study of Lung Cancer* (2018) 13(12):1906-18. Epub 2018/09/16. doi: 10.1016/j.jtho.2018.08.2027. PubMed PMID: 30217492.

6. Rittmeyer A, Barlesi F, Waterkamp D, Park K, Ciardiello F, von Pawel J, et al. Atezolizumab versus docetaxel in patients with previously treated non-small-cell lung cancer (OAK): a phase 3, open-label, multicentre randomised controlled trial. *Lancet (London, England)* (2017) 389(10066):255-65. Epub 2016/12/17. doi: 10.1016/s0140-6736(16)32517-x. PubMed PMID: 27979383; PubMed Central PMCID: PMCPMC6886121.

7. McDermott DF, Sosman JA, Sznol M, Massard C, Gordon MS, Hamid O, et al. Atezolizumab, an Anti-Programmed Death-Ligand 1 Antibody, in Metastatic Renal Cell Carcinoma: Long-Term Safety, Clinical Activity, and Immune Correlates From a Phase Ia Study. *Journal of clinical oncology : official journal of the American Society of Clinical Oncology* (2016) 34(8):833-42. Epub 2016/01/13. doi: 10.1200/jco.2015.63.7421. PubMed PMID: 26755520.

8. Heery CR, O'Sullivan-Coyne G, Madan RA, Cordes L, Rajan A, Rauckhorst M, et al. Avelumab for metastatic or locally advanced previously treated solid tumours (JAVELIN Solid Tumor): a phase 1a, multicohort, dose-escalation trial. *The Lancet Oncology* (2017) 18(5):587-98. Epub 2017/04/05. doi: 10.1016/s1470-2045(17)30239-5. PubMed PMID: 28373007; PubMed Central PMCID: PMCPMC6387686.

9. Gulley JL, Rajan A, Spigel DR, Iannotti N, Chandler J, Wong DJL, et al. Avelumab for patients with previously treated metastatic or recurrent non-small-cell lung cancer (JAVELIN Solid Tumor): dose-expansion cohort of a multicentre, open-label, phase 1b trial. *The Lancet Oncology* (2017) 18(5):599-610. Epub 2017/04/05. doi: 10.1016/s1470-2045(17)30240-1. PubMed PMID: 28373005; PubMed Central PMCID: PMCPMC5522719.

10. Kaufman HL, Russell J, Hamid O, Bhatia S, Terheyden P, D'Angelo SP, et al. Avelumab in patients with chemotherapy-refractory metastatic Merkel cell carcinoma: a multicentre, single-group, open-label, phase 2 trial. *The Lancet Oncology* (2016) 17(10):1374-85. Epub 2016/09/07. doi: 10.1016/s1470-2045(16)30364-3. PubMed PMID: 27592805; PubMed Central PMCID: PMCPMC5587154.

11. Le Tourneau C, Hoimes C, Zarwan C, Wong DJ, Bauer S, Claus R, et al. Avelumab in patients with previously treated metastatic adrenocortical carcinoma: phase 1b results from the JAVELIN solid tumor trial. *J Immunother Cancer* (2018) 6(1):111. Epub 2018/10/24. doi: 10.1186/s40425-018-0424-9. PubMed PMID: 30348224; PubMed Central PMCID: PMCPMC6198369.

12. Apolo AB, Infante JR, Balmanoukian A, Patel MR, Wang D, Kelly K, et al. Avelumab, an Anti-Programmed Death-Ligand 1 Antibody, In Patients With Refractory Metastatic Urothelial Carcinoma: Results From a Multicenter, Phase Ib Study. *Journal of clinical oncology : official journal of the American Society of Clinical Oncology* (2017) 35(19):2117-24. Epub 2017/04/05. doi: 10.1200/jco.2016.71.6795. PubMed PMID: 28375787; PubMed Central PMCID: PMCPMC5493051.

13. Lukas RV, Rodon J, Becker K, Wong ET, Shih K, Touat M, et al. Clinical activity and safety of atezolizumab in patients with recurrent glioblastoma. *Journal of neuro-oncology* (2018) 140(2):317-28. Epub 2018/08/04. doi: 10.1007/s11060-018-2955-9. PubMed PMID: 30073642.

14. Levy A, Massard C, Soria JC, Deutsch E. Concurrent irradiation with the anti-programmed cell death ligand-1 immune checkpoint blocker durvalumab: Single centre subset analysis from a phase 1/2 trial. *European journal of cancer (Oxford, England : 1990)* (2016) 68:156-62. Epub 2016/10/21. doi: 10.1016/j.ejca.2016.09.013. PubMed PMID: 27764686.

15. Rebelatto MC, Midha A, Mistry A, Sabalos C, Schechter N, Li X, et al. Development of a programmed cell death ligand-1 immunohistochemical assay validated for analysis of non-small cell lung cancer and head and neck squamous cell carcinoma. *Diagnostic pathology* (2016) 11(1):95. Epub 2016/10/09. doi: 10.1186/s13000-016-0545-8. PubMed PMID: 27717372; PubMed Central PMCID: PMCPMC5055695.

16. Paz-Ares L, Dvorkin M, Chen Y, Reinmuth N, Hotta K, Trukhin D, et al. Durvalumab plus platinum–etoposide versus platinum–etoposide in first-line treatment of extensive-stage small-cell lung cancer (CASPIAN): a randomised, controlled, open-label, phase 3 trial. *The Lancet* (2019) 394(10212):1929-39. doi: 10.1016/S0140-6736(19)32222-6.

17. Rotman J, Mom CH, Jordanova ES, de Gruijl TD, Kenter GG. 'DURVIT': a phase-I trial of single low-dose durvalumab (Medi4736) IntraTumourally injected in cervical cancer: safety, toxicity and effect on the primary tumour- and lymph node microenvironment. *BMC cancer* (2018) 18(1):888. Epub 2018/09/14. doi: 10.1186/s12885-018-4764-0. PubMed PMID: 30208866; PubMed Central PMCID: PMCPMC6134598.

18. D'Angelo SP, Hunger M, Brohl AS, Nghiem P, Bhatia S, Hamid O, et al. Early objective response to avelumab treatment is associated with improved overall survival in patients with metastatic Merkel cell carcinoma. *Cancer immunology, immunotherapy : CII* (2019) 68(4):609-18. Epub 2019/02/06. doi: 10.1007/s00262-018-02295-4. PubMed PMID: 30721341; PubMed Central PMCID: PMCPMC6447510.

19. D'Angelo SP, Russell J, Lebbe C, Chmielowski B, Gambichler T, Grob JJ, et al. Efficacy and Safety of First-line Avelumab Treatment in Patients With Stage IV Metastatic Merkel Cell Carcinoma: A Preplanned Interim Analysis of a Clinical Trial. *JAMA oncology* (2018) 4(9):e180077. Epub 2018/03/23. doi: 10.1001/jamaoncol.2018.0077. PubMed PMID: 29566106; PubMed Central PMCID: PMCPMC5885245.

20. Rajan A, Heery CR, Thomas A, Mammen AL, Perry S, O'Sullivan Coyne G, et al. Efficacy and tolerability of anti-programmed death-ligand 1 (PD-L1) antibody (Avelumab) treatment in advanced thymoma. *Journal for ImmunoTherapy of Cancer* (2019) 7(1). doi: 10.1186/s40425-019-0723-9.

21. Mignard X, Antoine M, Moro-Sibilot D, Dayen C, Mennecier B, Gervais R, et al. [IoNESCO trial: Immune neoajuvant therapy in early stage non-small cell lung cancer]. *Revue des maladies respiratoires* (2018) 35(9):983-8. Epub 2018/09/24. doi: 10.1016/j.rmr.2018.08.006. PubMed PMID: 30243521.

22. Yu Y, Lee NY. JAVELIN Head and Neck 100: a Phase III trial of avelumab and chemoradiation for locally advanced head and neck cancer. *Future oncology (London, England)* (2019) 15(7):687-94. Epub 2018/11/22. doi: 10.2217/fon-2018-0405. PubMed PMID: 30461306.

23. Moehler M, Ryu MH, Dvorkin M, Lee KW, Coskun HS, Wong R, et al. Maintenance avelumab versus continuation of first-line chemotherapy in gastric cancer: JAVELIN Gastric 100 study design. *Future oncology (London, England)* (2019) 15(6):567-77. Epub 2018/11/01. doi: 10.2217/fon-2018-0668. PubMed PMID: 30379568.

24. Necchi A, Giannatempo P, Raggi D, Mariani L, Colecchia M, Fare E, et al. An Open-label Randomized Phase 2 study of Durvalumab Alone or in Combination with Tremelimumab in Patients with Advanced Germ Cell Tumors (APACHE): Results from the First Planned Interim Analysis. *Eur Urol* (2019) 75(1):201-3. Epub 2018/09/24. doi: 10.1016/j.eururo.2018.09.010. PubMed PMID: 30243800.

25. Chih-Hsin Yang J, Shepherd FA, Kim DW, Lee GW, Lee JS, Chang GC, et al. Osimertinib Plus Durvalumab versus Osimertinib Monotherapy in EGFR T790M–Positive NSCLC following Previous EGFR TKI Therapy: CAURAL Brief Report. *Journal of Thoracic Oncology* (2019) 14(5):933-9. doi: 10.1016/j.jtho.2019.02.001.

26. Migden MR, Rischin D, Schmults CD, Guminski A, Hauschild A, Lewis KD, et al. PD-1 Blockade with Cemiplimab in Advanced Cutaneous Squamous-Cell Carcinoma. *The New England journal of medicine* (2018) 379(4):341-51. Epub 2018/06/05. doi: 10.1056/NEJMoa1805131. PubMed PMID: 29863979.

27. Doi T, Iwasa S, Muro K, Satoh T, Hironaka S, Esaki T, et al. Phase 1 trial of avelumab (anti-PD-L1) in Japanese patients with advanced solid tumors, including dose expansion in patients with gastric or gastroesophageal junction cancer: the JAVELIN Solid Tumor JPN trial. *Gastric Cancer* (2019) 22(4):817-27. Epub 2018/12/06. doi: 10.1007/s10120-018-0903-1. PubMed PMID: 30515672; PubMed Central PMCID: PMCPMC6570778.

28. Mizugaki H, Yamamoto N, Murakami H, Kenmotsu H, Fujiwara Y, Ishida Y, et al. Phase I dose-finding study of monotherapy with atezolizumab, an engineered immunoglobulin monoclonal antibody targeting PD-L1, in Japanese patients with advanced solid tumors. *Investigational new drugs* (2016) 34(5):596-603. Epub 2016/07/02. doi: 10.1007/s10637-016-0371-6. PubMed PMID: 27363843; PubMed Central PMCID: PMCPMC5007272.

29. Bahig H, Aubin F, Stagg J, Gologan O, Ballivy O, Bissada E, et al. Phase I/II trial of Durvalumab plus Tremelimumab and stereotactic body radiotherapy for metastatic head and neck carcinoma. *BMC cancer* (2019) 19(1):68. Epub 2019/01/16. doi: 10.1186/s12885-019-5266-4. PubMed PMID: 30642290; PubMed Central PMCID: PMCPMC6332607.

30. Chia S, Bedard PL, Hilton J, Amir E, Gelmon K, Goodwin R, et al. A Phase Ib Trial of Durvalumab in Combination with Trastuzumab in HER2-Positive Metastatic Breast Cancer (CCTG IND.229). *The oncologist* (2019) 24(11):1439-45. doi: 10.1634/theoncologist.2019-0321.

31. Mego M, Svetlovska D, Chovanec M, Rečkova M, Rejlekova K, Obertova J, et al. Phase II study of avelumab in multiple relapsed/refractory germ cell cancer. *Investigational new drugs* (2019) 37(4):748-54. doi: 10.1007/s10637-019-00805-4.

32. Bang YJ, Ruiz EY, Van Cutsem E, Lee KW, Wyrwicz L, Schenker M, et al. Phase III, randomised trial of avelumab versus physician's choice of chemotherapy as third-line treatment of patients with advanced gastric or gastro-oesophageal junction cancer: primary analysis of JAVELIN Gastric 300. *Annals of oncology : official journal of the European Society for Medical Oncology* (2018) 29(10):2052-60. Epub 2018/07/28. doi: 10.1093/annonc/mdy264. PubMed PMID: 30052729; PubMed Central PMCID: PMCPMC6225815.

33. Shemesh CS, Chanu P, Jamsen K, Wada R, Rossato G, Donaldson F, et al. Population pharmacokinetics, exposure-safety, and immunogenicity of atezolizumab in pediatric and young adult patients with cancer. *Journal for ImmunoTherapy of Cancer* (2019) 7(1). doi: 10.1186/s40425-019-0791-x.

34. Loibl S, Untch M, Burchardi N, Huober J, Sinn BV, Blohmer JU, et al. A randomised phase II study investigating durvalumab in addition to an anthracycline taxane-based neoadjuvant therapy in early triple negative breast cancer - clinical results and biomarker analysis of GeparNuevo study. *Annals of oncology : official journal of the European Society for Medical Oncology* (2019). Epub 2019/05/17. doi: 10.1093/annonc/mdz158. PubMed PMID: 31095287.

35. Pujol JL, Greillier L, Audigier-Valette C, Moro-Sibilot D, Uwer L, Hureaux J, et al. A Randomized Non-Comparative Phase II Study of Anti-Programmed Cell Death-Ligand 1 Atezolizumab or Chemotherapy as Second-Line Therapy in Patients With Small Cell Lung Cancer: Results From the IFCT-1603 Trial. *Journal of Thoracic Oncology* (2019) 14(5):903-13. doi: 10.1016/j.jtho.2019.01.008.

36. Colevas AD, Bahleda R, Braiteh F, Balmanoukian A, Brana I, Chau NG, et al. Safety and clinical activity of atezolizumab in head and neck cancer: results from a phase I trial. *Annals of oncology : official journal of the European Society for Medical Oncology* (2018) 29(11):2247-53. Epub 2018/09/17. doi: 10.1093/annonc/mdy411. PubMed PMID: 30219915.

37. Horn L, Gettinger SN, Gordon MS, Herbst RS, Gandhi L, Felip E, et al. Safety and clinical activity of atezolizumab monotherapy in metastatic non-small-cell lung cancer: final results from a phase I study. *European journal of cancer (Oxford, England : 1990)* (2018) 101:201-9. Epub 2018/08/05. doi: 10.1016/j.ejca.2018.06.031. PubMed PMID: 30077125.

38. Allen CT, Lee S, Norberg SM, Kovalovsky D, Ye H, Clavijo PE, et al. Safety and clinical activity of PD-L1 blockade in patients with aggressive recurrent respiratory papillomatosis. *J Immunother Cancer* (2019) 7(1):119. Epub 2019/05/06. doi: 10.1186/s40425-019-0603-3. PubMed PMID: 31053174; PubMed Central PMCID: PMCPMC6500000.

39. Lee JM, Cimino-Mathews A, Peer CJ, Zimmer A, Lipkowitz S, Annunziata CM, et al. Safety and Clinical Activity of the Programmed Death-Ligand 1 Inhibitor Durvalumab in Combination With Poly (ADP-Ribose) Polymerase Inhibitor Olaparib or Vascular Endothelial Growth Factor Receptor 1-3 Inhibitor Cediranib in Women's Cancers: A Dose-Escalation, Phase I Study. *Journal of clinical oncology : official journal of the American Society of Clinical Oncology* (2017) 35(19):2193-202. Epub 2017/05/05. doi: 10.1200/jco.2016.72.1340. PubMed PMID: 28471727; PubMed Central PMCID: PMCPMC5493052.

40. Massard C, Gordon MS, Sharma S, Rafii S, Wainberg ZA, Luke J, et al. Safety and Efficacy of Durvalumab (MEDI4736), an Anti-Programmed Cell Death Ligand-1 Immune Checkpoint Inhibitor, in Patients With Advanced Urothelial Bladder Cancer. *Journal of clinical oncology : official journal of the American Society of Clinical Oncology* (2016) 34(26):3119-25. Epub 2016/06/09. doi: 10.1200/jco.2016.67.9761. PubMed PMID: 27269937; PubMed Central PMCID: PMCPMC5569690.

41. Liu JF, Gordon M, Veneris J, Braiteh F, Balmanoukian A, Eder JP, et al. Safety, clinical activity and biomarker assessments of atezolizumab from a Phase I study in advanced/recurrent ovarian and uterine cancers. *Gynecol Oncol* (2019) 154(2):314-22. Epub 2019/06/18. doi: 10.1016/j.ygyno.2019.05.021. PubMed PMID: 31204078.

42. Fujiwara Y, Iguchi H, Yamamoto N, Hayama M, Nii M, Ueda S, et al. Tolerability and efficacy of durvalumab in Japanese patients with advanced solid tumors. *Cancer science* (2019) 110(5):1715-23. Epub 2019/03/21. doi: 10.1111/cas.14003. PubMed PMID: 30891877; PubMed Central PMCID: PMCPMC6501043.

43. Calabro L, Morra A, Giannarelli D, Amato G, D'Incecco A, Covre A, et al. Tremelimumab combined with durvalumab in patients with mesothelioma (NIBIT-MESO-1): an open-label, non-randomised, phase 2 study. *The Lancet Respiratory medicine* (2018) 6(6):451-60. Epub 2018/05/19. doi: 10.1016/s2213-2600(18)30151-6. PubMed PMID: 29773326.

44. Kaufman HL, Russell JS, Hamid O, Bhatia S, Terheyden P, D'Angelo SP, et al. Updated efficacy of avelumab in patients with previously treated metastatic Merkel cell carcinoma after >/=1 year of follow-up: JAVELIN Merkel 200, a phase 2 clinical trial. *J Immunother Cancer* (2018) 6(1):7. Epub 2018/01/20. doi: 10.1186/s40425-017-0310-x. PubMed PMID: 29347993; PubMed Central PMCID: PMCPMC5774167.
