# Supplementary material for: Fatal Adverse Events Associated With Programmed Cell Death Ligand 1 Inhibitors: A Systematic Review and Meta-Analysis
Source: Front Pharmacol. 2020 Jan 31;11:5. doi: 10.3389/fphar.2020.00005 (PMC7006642; doi:10.3389/fphar.2020.00005)
Supplement: Supplementary file 3 [file Table_3.doc]

**Supplement table 3. Characteristics of the studies showed in Figure 3**

| **Characteristics of the studies showed in Figure 3** | | | |
| --- | --- | --- | --- |
| **Author year** | **Cancer type** | **The No. in Figure 3** | **The No. in reference listn Reference** |
| Motzer RJ (2019) | Renal cell carcinoma | 1 | 23 |
| Powles T (2017) | Urothelial carcinoma | 2 | 28 |
| Antonia S (2016) | Non-small cell lung cancer | 3 | 34 |
| Antonia SJ (2017) | Non-small cell lung cancer | 4 | 26 |
| Balar AV (2017) | Urothelial carcinoma | 5 | 13 |
| Barlesi F (2018) | Non-small cell lung cancer | 6 | 5 |
| Choueiri TK (2018) | Renal cell carcinoma | 7 | 6 |
| Dirix LY (2018) | Breast cancer | 8 | 24 |
| Garassino MC (2018) | Non-small cell lung cancer | 9 | 27 |
| Horn L (2018) | Small cell lung cancer | 10 | 30 |
| Liu SV (2018) | Non-small cell lung cancer | 11 | 31 |
| Pal SK (2018) | Urothelial carcinoma | 12 | 16 |
| Patel MR (2018) | Urothelial carcinoma | 13 | 22 |
| Peters S (2017) | Non-small cell lung cancer | 14 | 33 |
| Powles T (2018) | Urothelial carcinoma | 15 | 18 |
| Schmid P (2018) | Breast cancer | 16 | 12 |
| Socinski MA (2018) | Non-squamous non-small cell  lung cancer | 17 | 14 |
| Rini BI (2019) | Renal cell carcinoma | 18 | 17 |
| West H (2019) | Non-squamous non-small cell  lung cancer | 19 | 15 |
| Hong D (2019) | Pancreatic cancer | 20 | 32 |
| Chung HC (2019) | Gastric or gastroesophageal  junction cancer | 21 | 21 |
| Spigel DR (2018) | Non-small cell lung cancer | 22 | 29 |
| Fehrenbacher L (2016) | Non-small cell lung cancer | 23 | 19 |
| Siu LL (2018) | Head and neck squamous  cell carcinoma | 24 | 35 |
| Eng C (2019) | Colorectal cancer | 25 | 20 |
| McDermott DF (2018) | Renal cell carcinoma | 26 | 25 |
